# Methylomarinovum tepidoasis sp. nov., a moderately thermophilic methanotroph of the family Methylothermaceae isolated from a deep-sea hydrothermal field

**DOI:** 10.1099/ijsem.0.006288

**Published:** 2024-03-13

**Authors:** Hisako Hirayama, Yoshihiro Takaki, Mariko Abe, Masayuki Miyazaki, Katsuyuki Uematsu, Yohei Matsui, Ken Takai

**Affiliations:** 1 Institute for Extra-cutting-edge Science and Technology Avant-garde Research (X-star), Japan Agency for Marine-Earth Science & Technology (JAMSTEC), Yokosuka, Kanagawa, Japan; 2 Marine Works Japan Ltd., Yokosuka, Kanagawa, Japan; 3 Research Institute for Global Change (RIGC), JAMSTEC, Yokosuka, Kanagawa, Japan

**Keywords:** deep-sea, hydrothermal, methane oxidation, methanotroph, Okinawa Trough, thermophilic

## Abstract

A novel aerobic methanotrophic bacterium, designated as strain IN45^T^, was isolated from *in situ* colonisation systems deployed at the Iheya North deep-sea hydrothermal field in the mid-Okinawa Trough. IN45^T^ was a moderately thermophilic obligate methanotroph that grew only on methane or methanol at temperatures between 25 and 56 °C (optimum 45–50 °C). It was an oval-shaped, Gram-reaction-negative, motile bacterium with a single polar flagellum and an intracytoplasmic membrane system. It required 1.5–4.0 % (w/v) NaCl (optimum 2–3 %) for growth. The major phospholipid fatty acids were C_16 : 1_ω7*c*, C_16 : 0_ and C_18 : 1_ω7*c*. The major isoprenoid quinone was Q-8. The 16S rRNA gene sequence comparison revealed 99.1 % sequence identity with *Methylomarinovum caldicuralii* IT-9^T^, the only species of the genus *Methylomarinovum* with a validly published name within the family *Methylothermaceae*. The complete genome sequence of IN45^T^ consisted of a 2.42-Mbp chromosome (DNA G+C content, 64.1 mol%) and a 20.5-kbp plasmid. The genome encodes genes for particulate methane monooxygenase and two types of methanol dehydrogenase (*mxaFI* and *xoxF*). Genes involved in the ribulose monophosphate pathway for carbon assimilation are encoded, but the transaldolase gene was not found. The genome indicated that IN45^T^ performs partial denitrification of nitrate to N_2_O, and its occurrence was indirectly confirmed by N_2_O production in cultures grown with nitrate. Genomic relatedness indices between the complete genome sequences of IN45^T^ and *M. caldicuralii* IT-9^T^, such as digital DNA–DNA hybridisation (51.2 %), average nucleotide identity (92.94 %) and average amino acid identity (93.21 %), indicated that these two methanotrophs should be separated at the species level. On the basis of these results, strain IN45^T^ represents a novel species, for which we propose the name *Methylomarinovum tepidoasis* sp. nov. with IN45^T^ (=JCM 35101^T^ =DSM 113422^T^) as the type strain.

## Introduction

Methane sources can be found in a variety of marine environments [1], among which deep-sea hydrothermal vent fields are attractive due to their ecological uniqueness [2]. The results of molecular studies have indicated that several deep-sea hydrothermal fields harbour diverse aerobic methanotrophic bacteria [3,7], whereas no methanotrophic isolates from deep-sea hydrothermal environments have been described. In contrast, from shallow submarine hydrothermal systems, moderately thermophilic or thermotolerant marine methanotrophs have been isolated and described. These are *Methylomarinovum caldicuralii* IT-9^T^ (growing at temperatures up to 55 °C) of the family *Methylothermaceae* from a water depth of 23 m [8] and *Methylocaldum marinum* S8^T^ (growing at temperatures up to 47 °C) of the family *Methylococcaceae* from a water depth of 161 m [9], both members of the order *Methylococcales* within the class Gammaproteobacteria.

The family *Methylothermaceae* currently consists of three genera (*Methylothermus*, *Methylohalobius* and *Methylomarinovum*) and four methanotrophic species, including two species of the genus *Methylothermus* isolated from terrestrial hot springs [10,11], one species of the genus *Methylohalobius* from a hypersaline lake [12] and one species of the genus *Methylomarinovum* (i.e., *M. caldicuralii* IT-9^T^) from a shallow submarine hydrothermal system in a coral reef area [8]. All four species are moderate thermophiles and/or slight or moderate halophiles; these physiological characteristics are typical of members of the family *Methylothermaceae*. As molecular signatures of members of the family *Methylothermaceae* have been detected in deep-sea hydrothermal fields, such as the particulate methane monooxygenase gene *pmoA* from the 13 °N East Pacific Rise [6] and the Rainbow field in the Mid-Atlantic Ridge [6], and a near-complete genome sequence from the southern Lau Basin [7], it is likely that deep-sea hydrothermal fields are also habitats for methanotrophs of the family *Methylothermaceae.*

We have studied methanotrophic bacteria at the Original site in the Iheya North deep-sea hydrothermal field in the mid-Okinawa Trough, Japan, and recently reported the presence of various methanotrophs of the order *Methylococcales* [5]. In a series of studies in this field, we successfully isolated a moderately thermophilic methanotroph, designated as strain IN45^T^, from deep-sea habitats at a depth of approximately 1000 m. Rush *et al*. [13] have analysed IN45^T^ previously for bacteriohopanepolyols, but it has not been described taxonomically. Herein, we characterised IN45^T^ as representing a novel species of the genus *Methylomarinovum* within the family *Methylothermaceae*.

## Methods

### Enrichment and isolation

To collect microorganisms from the Original site in the Iheya North deep-sea hydrothermal field, *in situ* colonisation systems (ISCSs) were deployed in chemosynthetic animal colonies for 2 months from November 2013 (cruise NT13-22) to January 2014 (cruise KY14-01) using the remotely operated vehicle *Hyper-Dolphin*. ISCS-1 and ISCS-4 were used in this cultivation experiment. These two ISCSs were deployed in colonies of the galatheoid crab *Shinkaia crosnieri* in hydrothermal diffuse-flow areas at depths of 1058 m (27 ° 47′ 25″ N, 126 ° 54′ 02″ E) and 986 m (27 ° 47′ 27″ N, 126 ° 53′ 48″ E), respectively. The deployment and recovery of ISCSs and a detailed description of the site have been reported previously [5].

A methane-fed continuous flowthrough cultivation system [5] was used to enrich methanotrophs from a mixture of ceramic particles in ISCS-1 and ISCS-4. This system provides methanotrophs with a constant supply of low concentrations of growth substrates and avoids the accumulation of excreted metabolites. Compared with a batch cultivation system, it may provide conditions more similar to natural habitats and may help deep-sea methanotrophs to acclimate to laboratory conditions. The cultivation system consisted of a medium bottle connected to a bag containing a mixture of 83.8 % N_2_, 15 % CH_4_ and 1.2 % O_2_, a peristaltic pump (Masterflex L/S 7550–50; Cole-Parmer) controlled by an electronic on–off timer, and a cultivation column. The culture medium was periodically supplied at a rate of 20 ml min^−1^ for 1 min, followed by a 1 h pause. The cultivation column was heated to 45 °C in the incubator. The medium (pH 6.8) was prepared using REI-SEA Marine seawater (IWAKI) supplemented with (per litre) 0.1 g NaHCO_3_, 29 mg NH_4_NO_3_, 5 mg Na_2_HPO_4_, 2 mg KH_2_PO_4_, 0.025 mg CuSO_4_·5H_2_O, and 1.6 ml DSMZ 141 trace element solution.

Cultivation was initiated in February 2014 using the methane-fed continuous flowthrough system, and an enrichment culture of methanotrophic bacteria was obtained in April 2014 (5 weeks after the start of cultivation). For isolation, a portion of the enrichment culture was transferred to 3 ml of MJmet medium [8]. The medium was prepared in a 15 ml glass test tube, and the final pH was adjusted to 6.4 and 6.8 with gas phases of 28 % CH_4_, 13 % CO_2_, 6 % O_2_ and 28 % CH_4_, 5 % CO_2_, 6 % O_2_ (N_2_ balance, 150 kPa), respectively. The inoculated test tubes were incubated at 45 °C with shaking at 120 r.p.m., and cell growth was observed at pH 6.4 after 1 week of incubation. The culture was purified by repeating the serial dilution-to-extinction technique at least four times, as described previously [10]. As a result, a methanotrophic bacterium, designated as IN45^T^, was isolated. Heterotrophic contamination was tested using a medium containing 0.1–1 % (w/v) yeast extract instead of methane, but no such contamination was detected. The purity of the isolate was confirmed by successful direct sequencing of the partial 16S rRNA gene at least three times in independent cultures. Unless otherwise specified, IN45^T^ was subsequently analysed by culturing under the same conditions as used for isolation.

### 16S rRNA gene analysis

Genomic DNA was extracted from IN45^T^ using a DNeasy UltraClean Microbial Kit (Qiagen). The 16S rRNA gene was amplified via PCR using Bac27F and U1492R primers [14], and the purified PCR products were directly Sanger-sequenced. The resulting sequence (1466 bp) was subjected to a blast search (https://blast.ncbi.nlm.nih.gov/Blast.cgi), and sequence similarity was further analysed using GENETYX-MAC version 21.0.1 (GENETYX). The 16S rRNA gene sequences of IN45^T^ and reference strains were aligned using sina [15] on the Silva website (https://www.arb-silva.de/). The alignment was corrected manually where necessary and ambiguous regions were deleted. A maximum likelihood phylogenetic tree was reconstructed using the IQ-TREE web server [16] with the substitution model of Tamura and Nei with empirical base frequencies, a proportion of invariable sites and the discrete gamma model with four rate categories (TN+F+I+G4) selected by ModelFinder [17] and ultrafast bootstrap analysis of 1000 replicates [18].

The 16S rRNA gene sequence has been deposited at DDBJ/EMBL/GenBank (accession number LC770110).

### Whole-genome analysis

The whole-genome sequences of IN45^T^ and *M. caldicuralii* IT-9^T^ were determined. A culture of *M. caldicuralii* IT-9^T^, which has been maintained in our laboratory since its isolation, was used for the analysis. Genomic DNA was extracted using a NucleoSpin Tissue kit (Macherey-Nagel). Sequencing was performed on the MiSeq (Illumina) and Sequel (PacBio) platforms. PacBio subreads were assembled using the HGAP4 pipeline [19] from the PacBio SMRT toolkit (SMRT Link v6.0.0). Subsequently, the contigs were extended and combined with Illumina reads using PRICE version 1.0 [20]. Finally, the assemblies were error-corrected using Pilon version 1.18 [21]. Protein-coding genes in both genomes were predicted using Prodigal version 2.6.3 [22]. Their functional annotations were assigned using the Kyoto Encyclopedia of Genes and Genomes (KEGG) orthology database as a reference. Noncoding RNA genes, such as rRNAs and tRNAs, were predicted using INFERNAL version 1.1.4 [23] and tRNAscan-SE version 1.3.1 [24].

To infer the taxonomic position of the isolate, overall genomic relatedness was assessed. Digital DNA–DNA hybridisation (dDDH) values were estimated using the Genome-to-Genome Distance Calculator 3.0 (GGDC; http://ggdc.dsmz.de/ggdc.php#) [25]. Average nucleotide identity (ANI) and average amino acid identity (AAI) were estimated using OrthoANI [26] and AAI calculator (http://enve-omics.ce.gatech.edu/aai/), respectively. A genome-based phylogenetic tree was reconstructed using a concatenated amino acid alignment of 96 single-copy marker genes identified using the Genome Taxonomy Database Toolkit [27]. A maximum likelihood phylogenomic tree was inferred using RAxML [28] with the Le and Gascuel four-matrix model fused with free-rate heterogeneity and gamma rate heterogeneity (LG4X+G) and 300 bootstrap replicates.

For phylogenetic analysis of PmoA, the complete PmoA sequence (254 amino acid positions) deduced from the genome of IN45^T^ was aligned with reference PmoA sequences using muscle [29]. A maximum likelihood phylogenetic tree was reconstructed using the IQ-TREE web server [16] with the substitution model Le and Gascuel with empirical base frequencies and the discrete gamma model with four rate categories (LG+F+G4) selected by ModelFinder [17] and ultrafast bootstrap analysis of 1000 replicates [18].

The genome sequences have been deposited at DDBJ/EMBL/GenBank under accession numbers AP024718 and AP024719 for the chromosome and plasmid of IN45^T^, respectively, and AP024714 for the genome of *M. caldicuralii* IT-9^T^.

### Morphological analysis

Cells were routinely observed by phase-contrast microscopy using a BX51 microscope (Olympus). The Gram reaction of cells was determined using the KOH method [30]. The presence of flagella was determined by negative staining of cells with 1 % (w/v) neutral phosphotungstic acid. Ultrathin sections of cells were prepared by modifying a method reported previously [31]. Briefly, cells at the exponential growth phase were fixed with 2.5 % (w/v) glutaraldehyde and then subjected to high-pressure freezing (EM-PACT2, Leica) and freeze substitution. To enhance the contrast of intracellular organelles, the samples were *en bloc* stained via sequential treatment with osmium tetroxide and thiocarbohydrazide (OTO staining) [32] prior to dehydration and then embedded in epoxy resin. Transmission electron microscopy was performed under a Tecnai 20 electron microscope (FEI/Thermo Fisher Scientific) operating at 120 kV.

### Physiological analysis

Growth conditions were examined using MJmet medium, which was modified as necessary. For the pH range test, media with different pH values were prepared by adding HCl or NaOH. Growth on solid media was assessed at 37 °C using MJmet medium solidified at a slant with 0.8 % gellan gum (Nacalai Tesque) or 1.5 % Noble agar (Difco) in a 100 ml vial with a butyl rubber cap. Before solidification, each solidifying agent was autoclaved in distilled water and then combined with an appropriate concentration of MJmet medium. The final pH of the solidified media was adjusted from 6.0 to 6.3. The gas phase of the vial was prepared as 16–23 % CH_4_, 5–10 % CO_2_ and 5–6 % O_2_ (N_2_ balance, 120–130 kPa).

Growth on carbon substrates other than methane was examined by replacing methane with N_2_. Methanol was evaluated at a concentration range of 0.1–6 % (v/v). The following carbon substrates were also tested: 0.05 % (w/v) formate, acetate, citrate, succinate, glucose, fructose, ribose, mannitol, methylamine, dimethylamine and ethanol as well as 0.1 % (w/v) yeast extract and casamino acids. Nitrogen sources for growth were examined using MJmet medium prepared without nitrogen compounds as the base medium. The following substrates were tested: 0.05 and 0.1 % (w/v) casamino acids and 0.05 % (w/v) NH_4_Cl, NaNO_3_, NaNO_2_, urea, Tris, methylamine, dimethylamine and l-aspartate.

N_2_O and O_2_ were analysed using cultures (3 ml of medium in a 15 ml tube) grown under low-oxygen (1 % O_2_, 140 kPa) or normal-oxygen (7 % O_2_, 140 kPa) conditions. The headspace gas (0.2 ml for N_2_O and 0.3 ml for O_2_) in the culture tubes was analysed using a 7890A GC system (Agilent Technologies) equipped with a thermal conductivity detector. The analysis of N_2_O was performed on a ShinCarbon ST micropacked column (100/120 mesh, 2 m×1 mm internal diameter, Restek) with a helium carrier at a flow rate of 7.0 ml min^−1^ and an oven temperature programme as follows: 100 °C for 2 min, ramp at 15 °C min^−1^ to 300 °C and hold for 6 min. The analysis of O_2_ was performed on a Molesieve 5A micropacked column (80/100 mesh, 2 m×1 mm internal diameter, Restek) with an argon carrier at a flow rate of 7.0 ml min^−1^ and an oven temperature programme as follows: 30 °C for 6.5 min, ramp at 60 °C min^−1^ to 120 °C and hold for 2 min.

### Chemotaxonomic analysis

Isoprenoid quinones, polar lipids and whole-cell fatty acids were analysed using exponentially grown cells. Isoprenoid quinones and polar lipids were extracted from lyophilised cells using methods described previously [33]. Isoprenoid quinones were purified by thin-layer chromatography and analysed using HPLC [34]. Polar lipids were determined using two-dimensional thin-layer chromatography [33,34]. Fatty acid methyl esters were prepared following the Sherlock Microbial Identification System protocol [35]. To determine the double bond positions, a portion of the fatty acid methyl esters was derivatised with dimethyl disulphide [36]. Fatty acid methyl esters were analysed on a JMS-Q1500GC GC-MS system (JEOL) using two capillary columns of different polarity. The first analysis was performed on a Supelco SP-2560 column (100 m×0.25 mm internal diameter, 0.20 µm film thickness) at a helium carrier flow rate of 1.0 ml min^−1^ with an oven temperature programme of 160–240 °C (2 °C min^−1^) and hold time of 15 min. The second analysis was performed on an Agilent DB-5MS column (30 m×0.25 mm internal diameter, 0.25 µm film thickness) at a helium flow rate of 1.5 ml min^−1^ with an oven temperature programme of 120–280 °C (320 °C for dimethyl disulphide derivatives, 4 °C min^−1^) and hold time of 5 min. All data were combined, and the fatty acid composition was calculated.

## Results and discussion

### Genetic identification of IN45^T^

**Fig. 1.**
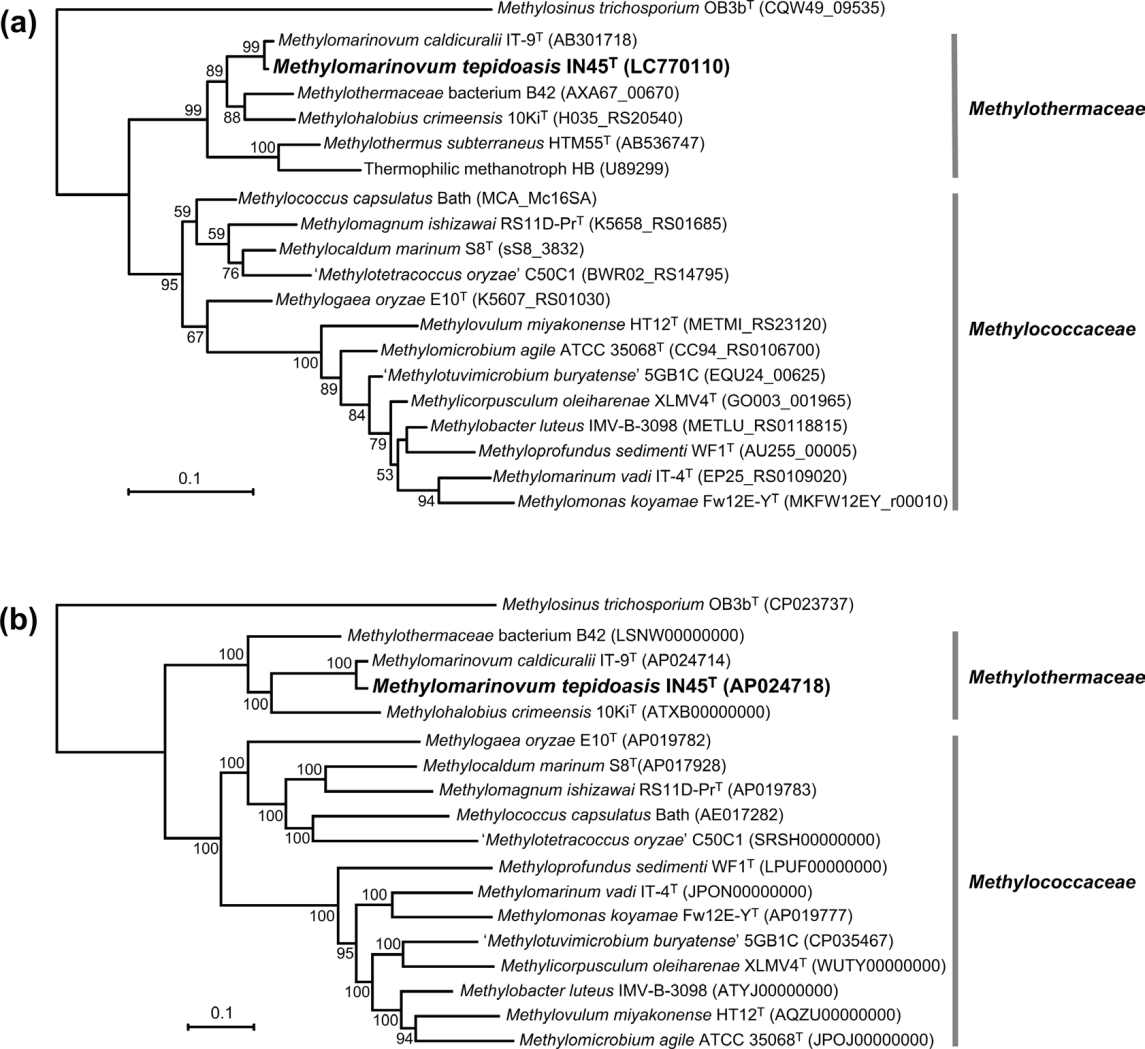
Phylogenetic position of strain IN45^T^ among reference species of the families *Methylothermaceae* and *Methylococcaceae*. (**a**) Maximum likelihood phylogenetic tree based on 16S rRNA gene sequences (1393 nucleotide positions). Bootstrap values >50 % are indicated at nodes. (**b**) Phylogenomic tree based on the concatenated amino acid sequences of 96 single-copy marker genes. The reference strains with available high-quality genomes were used in the analysis. Accession numbers or gene locus tags are shown in parentheses. *Methylosinus trichosporium* OB3b^T^ was used as an outgroup species.

A methanotrophic enrichment culture was obtained from ISCSs deployed in chemosynthetic animal colonies in the Iheya North deep-sea hydrothermal field, and from which a methanotrophic bacterium, designated as IN45^T^, was isolated at 45 °C. The 16S rRNA gene sequence (1466 bp) was obtained for IN45^T^ via PCR amplification and direct sequencing. Sequence comparison revealed that IN45^T^ was closely related to *M. caldicuralii* IT-9^T^ within the family *Methylothermaceae*, with a sequence identity of 99.1 %. This value was above the proposed species boundary cut-off (98.65 %) [37]. IN45^T^ was moderately related to other members of the family *Methylothermaceae*, including *Methylohalobius crimeensis* 10Ki^T^ (94.7 % identity), species of the genus *Methylothermus* (91.4–91.6 %) and the uncultured bacterium B42 identified from a deep-sea hydrothermal vent (94.6 %) [7] The phylogenetic tree of 16S rRNA gene sequences is shown in Fig. 1a.

Owing to the high degree of similarity between the 16S rRNA gene sequences of IN45^T^ and *M. caldicuralii* IT-9^T^, it was necessary to compare their genomes in order to assign a taxonomic position to IN45^T^. Accordingly, we performed whole-genome sequencing of IN45^T^. In addition, the reference bacterium *M. caldicuralii* IT-9^T^, for which no genome sequence was available, was analysed. As a result, the complete genome sequences of IN45^T^ and *M. caldicuralii* IT-9^T^ were obtained. The genome of IN45^T^ was reconstructed as a 2.42 Mbp chromosome and a 20.5 kbp plasmid, and the chromosomal DNA G+C content was 64.1 mol%. The *M. caldicuralii* IT-9^T^ genome was 2.69 Mbp in size, with a DNA G+C content of 64.6 mol%. The genome features are summarised in Table 1.

**Table 1.** Summary of genomic and phenotypic features of strain IN45^T^ and a related species of the genus *Methylomarinovum* Strains: 1, IN45^T^ (data from this study); 2, *Methylomarinovum caldicuralii* IT-9^T^ (data from this study and from 8); +, Present or positive; −, absent or negative; pMMO, particulate methane monooxygenase; sMMO, soluble methane monooxygenase; RuMP, ribulose monophosphate; RuBisCO, ribulose-1,5-bisphosphate carboxylase; PS, phosphatidylserine; APL, unknown aminophospholipid; PL, unknown phospholipid; Q-8, ubiquinone 8.

Characteristic	1	2
Genome information:		
Genome accession numbers	AP024718, AP024719	AP024714
Assembly status	Complete	Complete
Number of contigs	2 (Chromosome, 1; Plasmid, 1)	1
Chromosome size (bp)	2 421 873	2 694 844
Plasmid size (bp)	20 514	−
Chromosome DNA G＋C content (mol%)	64.1	64.6
Plasmid DNA G＋C content (mol%)	58.7	−
Genome coverage	1058×	870×
Number of protein coding genes	2380	2614
Number of rRNA genes (16S, 23S, 5S)	2, 2, 2	2, 2, 2
Number of tRNA genes	46	47
Presence of genes for:		
pMMO	+	+
sMMO	−	−
MxaFI methanol dehydrogenase	+	+
XoxF methanol dehydrogenase	+	+
Nitrogen fixation	−	−
Hydroxylamine dehydrogenase	+	+
Dissimilatory nitrate reductase	+	−
Assimilatory nitrate reductase	+	−
RuMP pathway	+	+
Serine pathway	−	−
RuBisCO	−	−
Hemerythrin	+	−
Growth conditions:		
Temperature (optimum) (°C)	25–­56 (45–50)	30–55 (45–50)
pH (optimum)	5.2–6.9 (5.9–6.4)	5.3–6.9 (6.0–6.4)
NaCl concentration (optimum) (%, w/v)	1.5–4 (2–3)	1–5 (3)
Nitrogen sources	Ammonium, Nitrate	Ammonium, Urea
Vitamin requirement	+	−
Cell morphology (cell size, µm)	Ovoids (1.0–3.0×0.8–1.5)	Ovoids or Cocci (0.9–1.5×0.6–1.3)
Motility	+ (polar flagellum)	+ (polar flagellum)
Major fatty acids (>10 %)	C_16 : 1_ω7*c*, C_16 : 0_, C_18 : 1_ω7*c*	C_16 : 0_, C_18 : 1_ω7*c*
Major polar lipids	PS	APL, PL
Major quinone	Q-8	Q-8

Overall genomic relatedness based on dDDH, ANI and AAI was determined between IN45^T^ and *M. caldicuralii* IT-9^T^. The dDDH value was 51.2 % and the probability of a dDDH value ≥70 % was 22.5 % (formula 2). The ANI and AAI values were 92.94 and 93.21 %, respectively. All these values were below the proposed thresholds for species delineation (70 % for dDDH, 95–96 % for ANI, and 95 % for AAI) [25,38,40], indicating that IN45^T^ is distinct from *M. caldicuralii* IT-9^T^ at the species level. In the genome-based phylogenetic tree, IN45^T^ was found to cluster with *M. caldicuralii* IT-9^T^ within the family *Methylothermaceae* (Fig. 1b). The phylogenetic tree of deduced PmoA sequences also showed a clustering of these two strains within this family (Fig. S1, available in the online version of this article).

### Genomic characteristics of IN45^T^

The genome sequence indicated that IN45^T^ possesses key genes and pathways for methane metabolism (Table 1). Selected key genes are listed in Table S1. For methane oxidation, the genome contains two copies of the *pmoCAB* gene cluster and two orphan *pmoC* encoding particulate methane monooxygenase, but no soluble methane monooxygenase genes or *pxmABC* for another membrane-bound monooxygenase. For methanol oxidation, the genome contains one copy each of *mxaFI* and *xoxF* (clade 5), which encode different types of methanol dehydrogenases, and genes involved in the synthesis of the redox cofactor pyrroloquinoline quinone (*pqqABCDE*). The genes involved in the following pathways are also encoded: the tetrahydromethanopterin-mediated and tetrahydrofolate-mediated C_1_ transfer pathways, the ribulose monophosphate (RuMP) pathway for carbon assimilation, the Embden–Meyerhof–Parnas (EMP) glycolytic pathway, the tricarboxylic acid cycle and the aerobic respiratory chain. However, the serine pathway is incomplete, and the Calvin–Benson–Basham cycle and the Entner–Doudoroff glycolytic pathway are absent. Glycogen synthesis genes (*glgABC*) are encoded, indicating that the strain stores carbon as glycogen. A homologue of hemerythrin, a nonheme iron protein thought to transport oxygen, is encoded by the genome. Cytoplasmic hemerythrin has been reported to enhance the activity of particulate methane monooxygenase [41] and/or aerobic respiration [42] in methanotrophs, although the overall molecular mechanisms are not fully understood.

**Fig. 2.**
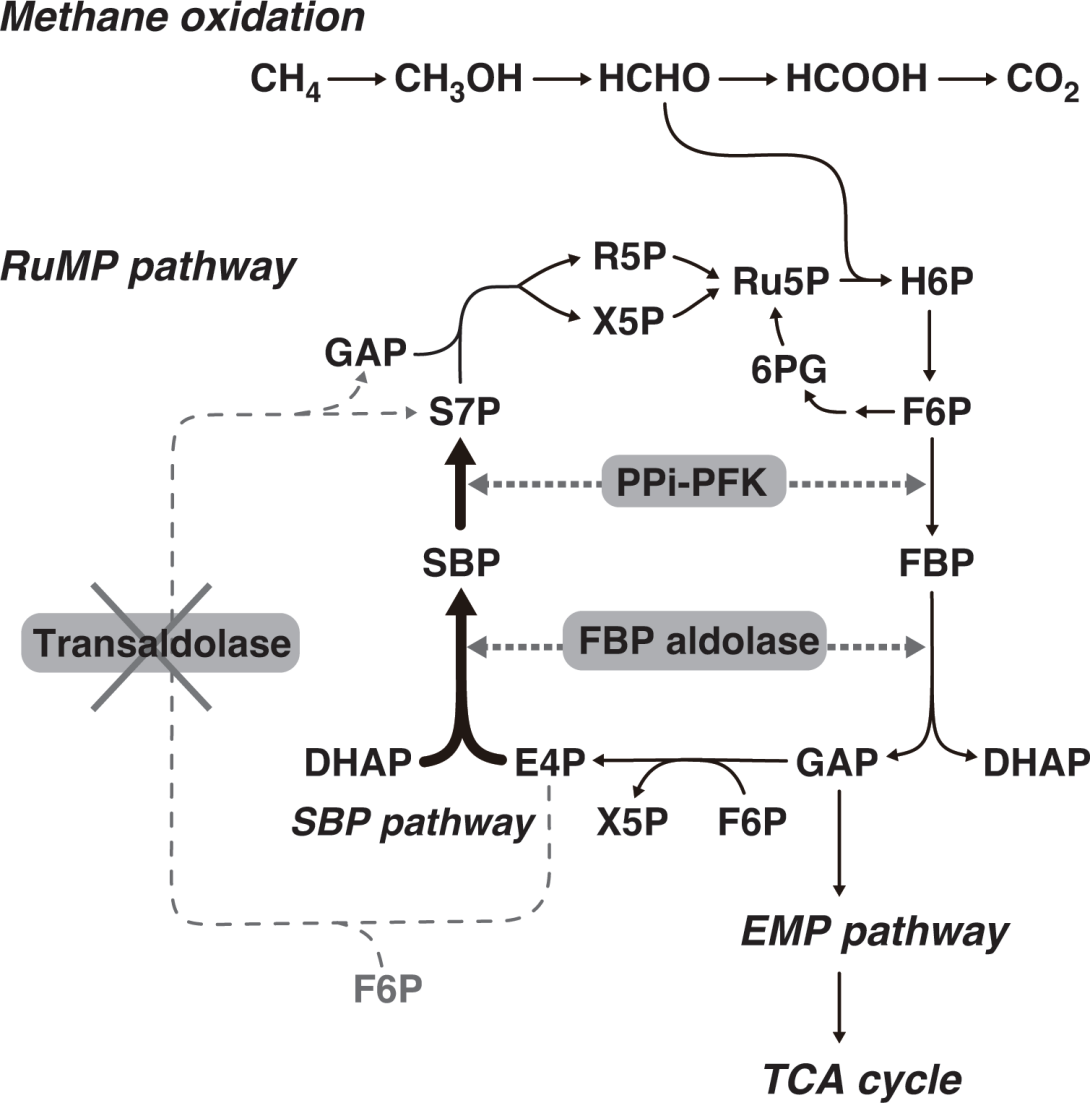
Simplified scheme of central carbon metabolism in IN45^T^. Thin dashed arrows indicate a reaction of transaldolase. In IN45^T^, transaldolase is absent and its absence can be compensated for by the sedoheptulose 1,7-bisphosphate (SBP) pathway (bold unbroken arrows). The SBP pathway can be catalysed by two canonical ribulose monophosphate (RuMP) pathway enzymes, fructose 1,6-bisphosphate (FBP) aldolase and pyrophosphate-dependent 6-phosphofructokinase (PPi-PFK). Abbreviations: Ru5P, ribulose 5-phosphate; H6P, 3-hexulose 6-phosphate; F6P, fructose 6-phosphate; 6 PG, 6-phosphogluconate; DHAP, dihydroxyacetone phosphate; GAP, glyceraldehyde 3-phosphate; E4P, erythrose 4-phosphate; X5P, xylulose 5-phosphate; S7P, sedoheptulose 7-phosphate; R5P, ribose 5-phosphate; EMP, Embden–Meyerhof–Parnas; TCA, tricarboxylic acid.

As described above, IN45^T^ will assimilate carbon via the RuMP pathway. Notably, the transaldolase gene was not found in the IN45^T^ genome. Transaldolase produces sedoheptulose 7-phosphate (S7P) as an obligate intermediate in the RuMP pathway and is also generally involved in the pentose phosphate pathway. However, despite the absence of the transaldolase gene, IN45^T^ is likely to produce S7P via the sedoheptulose 1,7-bisphosphate (SBP) pathway [43], which is essentially identical to part of the Calvin–Benson–Basham cycle. Two canonical RuMP pathway enzymes, fructose-1,6-bisphosphate aldolase and pyrophosphate-dependent 6-phosphofructokinase, can catalyse two reactions in the SBP pathway (Fig. 2). The reactions are as follows: fructose-1,6-bisphosphate aldolase forms SBP by condensation of erythrose 4-phosphate and dihydroxyacetone phosphate, and then pyrophosphate-dependent 6-phosphofructokinase dephosphorylates SBP to form S7P (Fig. 2). In addition to the canonical reactions with fructose phosphates, these two enzymes have been shown to catalyse the above-mentioned reactions with sedoheptulose phosphates (regardless of the direction of the reactions) in different bacteria, including methanotrophs [43,49]. Furthermore, it has been reported that the SBP pathway is feasible or likely to have evolved as part of the RuMP or pentose phosphate pathways in several bacteria, including native [50] and synthetic [51,52] methylotrophs and bacteria assimilating pentose sugars without the transaldolase gene [43,44, 46].

Regarding nitrogen metabolism, IN45^T^ carries genes for ammonium assimilation (*glnA* and *GDH2*), nitrate assimilation (*nasA* and *nirBD*), partial denitrification of nitrate to N_2_O (*narGHJI*, *nirK*, and *norBC*) and hydroxylamine dehydrogenase (*hao*). This is consistent with the finding that IN45^T^ used both ammonium and nitrate as the sole nitrogen source and that the strain produced N_2_O in the presence of nitrate, as described later.

Other genes of interest in IN45^T^ are homologues of *bcsABZC* for cellulose synthesis. These genes have been found in some gammaproteobacterial methanotrophic strains, but are less common in methanotrophs [5]. Cellulose is believed to play a role in the survival strategies of some cellulose-producing bacteria in terms of adhesion, colonisation and self-protection [53,54]. Thus, the ability to produce cellulose could be advantageous for the survival of microorganisms in deep-sea hydrothermal fields, where physicochemical conditions are highly variable.

### Genomic characteristics of *M. caldicuralii* IT-9^T^

In this study, the genome of the reference species *M. caldicuralii* IT-9^T^ was analysed and its genomic features are briefly summarised in Table 1. A list of key genes is given in Table S1. The genome revealed a high similarity between *M. caldicuralii* IT-9^T^ and IN45^T^ with respect to central carbon metabolism. The striking similarity is the unusual absence of transaldolase in the RuMP pathway, indicating that the alternative SBP pathway functions similarly to that in IN45^T^ (Fig. 2). In contrast, the nitrogen metabolism of the two strains differs significantly. *M. caldicuralii* IT-9^T^ lacks all genes for nitrate assimilation and denitrification. This is consistent with its inability to grow on nitrate when used as the sole nitrogen source [8]. Another notable feature is the absence of a homologue of the oxygen carrier hemerythrin despite its prevalence in many methanotrophs, including IN45^T^. Therefore, the absence of hemerythrin combined with the absence of denitrification enzymes in *M. caldicuralii* IT-9^T^ may reduce its metabolic activity under low-oxygen conditions. Intriguingly, similarly to IN45^T^, *M. caldicuralii* IT-9^T^ possesses *bcsABZC* homologues; however, *M. caldicuralii* IT-9^T^ most probably cannot synthesise cellulose because the *bcsA* homologue is disrupted by transposon insertion.

### Morphology

**Fig. 3.**
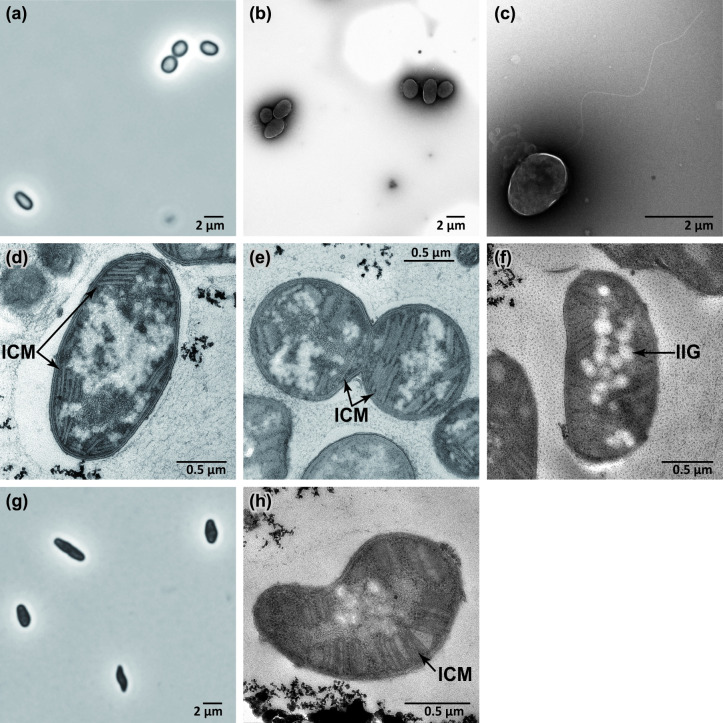
Images of cells of IN45^T^ obtained by phase-contrast microscopy (**a, g**) and transmission electron microscopy (b–f, **h**). Using transmission electron microscopy, negatively stained cells (**b, c**) and ultrathin sections of cells (d–f, **h**) were observed. Panels (**a**) – (**f**) show the cells grown at pH 6.1 (optimum pH for growth). Panels (**g**) and (**h**) show the cells grown at pH 6.7, where most of the cells were deformed. ICM, intracytoplasmic membranes; IIG, intracellular inclusions of granules.

Cells of IN45^T^ were Gram-reaction-negative and predominantly oval, partly coccoid or plump rod-shaped under optimum pH conditions (Fig. 3a–c). At pH 6.7 and above, most cells showed a deformed morphology (Fig. 3g, h). Cell size was approximately 1.0–3.0 µm long and 0.8–1.5 µm wide. Cells were motile with a single polar flagellum (Fig. 3c) and contained type I intracytoplasmic membranes and inclusion granules (Fig. 3d–f, h). In some cells of IN45^T^, the bundles of intracytoplasmic membranes showed a somewhat disordered or sparse arrangement (Fig. 3d), and a similar membrane arrangement has been reported in *M. caldicuralii* IT-9^T^ [8]. No cyst-like cells were observed in the culture after 1 month of storage.

### Physiology

IN45^T^ grew at temperatures between 25 and 56 °C (optimum 45–50 °C), but not at 23 °C or 57 °C. When a culture that had been stored for a few weeks or longer was used as inoculum, the two-step incubation, first at 37 °C for 1 day and then at 45 °C, appeared to shorten the growth lag. The strain grew at a pH range 5.2–6.9 (optimum pH 5.9–6.4) but not at pH 5.0 or 7.0. It seems strange that the strain could not grow at pH 7 or higher, since onboard measurements recorded pH 7.5 for fluid samples collected at the site of ISCS-4, one of the sources of isolation [5]. However, high-temperature hydrothermal fluids have been reported to be acidic (pH ≤5) [55], and mixing them with slightly alkaline cold seawater would create a gradient of environmental factors (temperature, pH and concentrations of substances, etc.) in the microbial habitats of this hydrothermal field. NaCl was required for growth at a concentration range 1.5–4 % (w/v) (optimum 2–3 %). Growth was not stable with 1 % NaCl and was not observed with 0.5 % or 5 % NaCl. At the time of isolation, Balch’s vitamin mixture [56] was routinely added to the medium, and the results of subsequent tests indicated that the strain required the vitamin mixture for growth. Under optimum conditions, IN45^T^ exhibited a maximum specific growth rate of 0.13–0.18 h^–1^ (doubling time of 4.0–5.4 h, *n*=3) and a maximum cell density of approximately 1–2×10^8^ cells ml^–1^.

Methanol supported the growth of IN45^T^ instead of methane at concentrations of 0.1–5 % (v/v), but not at 6 %. Neither formate nor any of the multicarbon substrates tested supported its growth. In the nitrogen source test, active growth was observed on NH_4_Cl and NaNO_3_. Although weak growth on urea was observed, no urease genes were found in the genome, so the strain may not have actually used urea for growth. Decomposition of urea in aqueous solution, where the product is generally ammonia, has been reported to occur over a wide temperature range, not only at high temperatures [57], and the possibility that some of the urea decomposed during incubation cannot be excluded. No apparent growth was observed on NaNO_2_, Tris, methylamine, dimethylamine, l-aspartate or casamino acids at the concentrations tested. In addition, gaseous N_2_ did not support growth. The growth test on solid media was performed three times independently; however, the strain did not form visible colonies on solid media, gellan gum or Noble agar, during incubation for 3 weeks.

As stated earlier, the genome of IN45^T^ encodes genes for partial denitrification of nitrate to N_2_O. We thus examined N_2_O production in batch cultures grown to stationary phase with or without NaNO_3_ supplementation (0.04 %, w/v). NH_4_Cl was added as nitrogen source. When IN45^T^ was grown with NaNO_3_ under low-oxygen conditions (1 % of the initial O_2_ concentration, no gas exchange during cultivation), N_2_O was detected in the headspace gas at concentrations of 21–126 ppm (average 81 ppm, *n*=4). In contrast, N_2_O concentrations in cultures without NaNO_3_ were low and below the limit of quantification (<5 ppm, *n*=4). During cultivation, O_2_ decreased from 1 % to 0.1–0.2 %. The medium with NaNO_3_ but not inoculated with cells (negative control) contained no detectable amounts of N_2_O (<2 ppm, *n*=2). This indicates that most of the N_2_O was produced from nitrate via denitrification, with a small amount from ammonium. IN45^T^ carries the *hao* gene, so the small amount of N_2_O was probably produced from ammonium via nitrifier denitrification, although this was not investigated in this study.

Under low-oxygen conditions, the final cell density of stationary-phase cultures was 2–3×10^7^ cells ml^–1^, which was several to 10 times lower than the density under normal oxygen conditions (1–2×10^8^ cells ml^–1^, 7 % O_2_). Even under normal-oxygen conditions, N_2_O was detected, probably due to the decreased oxygen levels (<2 % O_2_) in stationary-phase cultures; however, the N_2_O levels per cell were several times lower than those under low-oxygen conditions (data not shown). This is consistent with the general knowledge that denitrification is promoted under oxygen limitation [58]. The cell yield per ml oxygen was roughly estimated to be in the range of 3–4×10^8^ cells ml^−1^ oxygen, irrespective of low- or normal-oxygen conditions.

Partial denitrification of nitrate and N_2_O production have been reported in methanotrophic strains, particularly in members of the genus *Methylomonas*, including *Methylomonas methanica*, *Methylomonas koyamae* and *Methylomonas lenta* [59]. Furthermore, ‘*Methylomonas denitrificans*’ FJG1 has been shown to couple partial denitrification of nitrate and methane oxidation and to increase intracellular ATP levels under oxygen limitation [60]. The genes required for partial denitrification of nitrate are also present in some other proteobacterial methanotrophs [5]. Whether IN45^T^ can promote its growth or conserve cellular energy under oxygen limitation via denitrification remains to be investigated . If it can, the properties of this strain would be advantageous for survival in nature, as methane is generally abundant in anoxic environments [1].

### Chemotaxonomy

The major isoprenoid quinone in strain IN45^T^ was ubiquinone 8 (Q-8), representing 96.3 % of the quinone profile. The minor quinones detected were ubiquinone 7 (3.1 %), ubiquinone 6 (0.5 %) and ubiquinone 9 (0.1 %). Q-8 is the predominant quinone shared by members of the family *Methylothermaceae*, *M. caldicuralii* IT-9^T^ [8] and '*Methylothermus thermalis*' MYHT [11]. The major polar lipid in IN45^T^ was phosphatidylserine, while phosphatidylethanolamine, diphosphatidylglycerol, phosphatidylglycerol and several unknown phospholipids were also detected (Fig. S2). The polar lipid profile of IN45^T^ differed markedly from that of *M. caldicuralii* IT-9^T^, in which unknown phospholipids were detected as the major polar lipids [8].

The fatty acid compositions of IN45^T^ and other members of the family *Methylothermaceae* are shown in Table 2. The major fatty acids of IN45^T^ were C_16 : 1_ω7*c*, C_16 : 0_ and C_18 : 1_ω7*c*, which accounted for 94 % of the total fatty acids. The double bond positions of C_16 : 1_*ω*7*c* and C_18 : 1_ω7*c* were determined by analysis of dimethyl disulphide derivatives of these methyl esters. The GC–MS chromatogram and mass spectra of the dimethyl disulphide derivatives are shown in Fig. S3. The fatty acid profile of IN45^T^ was similar to that of *M. caldicuralii* IT-9^T^; however, the abundance of C_16 : 1_ω7*c* in IN45^T^ allowed the two strains to be distinguished. A relatively high abundance of C_16 : 1_ω7*c* (14.2–19.6 %) has also been reported in *Methylohalobius crimeensis*, another member of the family *Methylothermaceae* [12]. Currently, a common feature of the family members is the relatively high abundance of C_18 : 1_ species (28.6–60.5 %).

**Table 2.** Cellular fatty acid compositions of IN45^T^ and reference strains of members of the family *Methylothermaceae* Strains: 1, IN45^T^ (data from this study); 2, *Methylomarinovum caldicuralii* IT-9^T^ (data from 8); 3, *Methylohalobius crimeensis* 10Ki^T^ and 4Kr (data from 12); 4, '*Methylothermus thermalis*' MYHT (data from 11); 5, *Methylothermus subterraneus* HTM55^T^ (data from 10).

Fatty acid	1	2	3	4	5
C_10 : 0_	–	0.3	–	–	–
C_12 : 0_	–	0.4	0.2	–	0.5
C_14 : 0_	5.2	1.6	1.4–2.5	1.2	0.8
C_15 : 0_	0.4	0.3	0.3–0.5	2.1	0.6
C_16 : 1_ω9*c*	–	0.2	–	–	–
C_16 : 1_ω7*c*	32.4	8.0	14.2–19.6	3.5	2.2
C_16 : 1_ω7*t*＋ω5*c*	0.4	0.6	–	–	–
C_16 : 0_	32.9	43.0	22.8–23.0	37.2	52.0
C_16 : 0_ 2-OH	–	4.8	–	8.4	–
C_17 : 0_cyclo	–	–	0.7	4.7	1.7
C_17 : 0_	–	0.3	0.3	2.5	1.0
C_18 : 1_ω9*c*	–	–	–	35.2	­–
C_18 : 1_ω7*c*	28.6	39.1	51.9–60.5	0.4	34.8
C_18 : 1_ω7*t*	–	0.5	–	–	–
C_18 : 0_	0.1	1.0	0.5–0.6	1.7	4.0
C_19 : 0_cyclo	–	–	–	2.4	–
C_19 : 1_	–	–	–	–	2.5

### Proposal of a novel species of the genus *Methylomarinovum*

On the basis of its morphological, physiological, chemotaxonomic and phylogenetic characteristics, IN45^T^ represents a member of the genus *Methylomarinovum* within the family *Methylothermaceae*. The only species in this genus with a validly published name is *M. caldicuralii* IT-9^T^. The results indicate a high degree of similarity between IN45^T^ and *M. caldicuralii* IT-9^T^ in key taxonomic characteristics (Table 1), including growth conditions (temperature, pH and NaCl concentration), major fatty acid species, chromosomal DNA G+C content and 16S rRNA gene sequence. Furthermore, their basic carbon metabolism is similar, as indicated by the results of genome analysis.

However, a number of other characteristics distinguish IN45^T^ from *M. caldicuralii* IT-9^T^ (Table 1). Their major polar lipids were clearly different. Only IN45^T^ assimilated nitrate and required vitamins for growth. Only IN45^T^ carries genes for partial denitrification of nitrate, and the observed N_2_O production indicates that IN45^T^ can perform partial denitrification under oxygen limitation. A hemerythrin homologue was found in IN45^T^ but not in *M. caldicuralii* IT-9^T^. Furthermore, the overall genomic relatedness indices (dDDH, ANI and AAI) indicated that these two methanotrophs should be separated at the species level. In conclusion, strain IN45^T^ represents a novel species of the genus *Methylomarinovum*, for which we propose the name *Methylomarinovum tepidoasis* sp. nov.

## Description of *Methylomarinovum tepidoasis* sp. nov

*Methylomarinovum tepidoasis* (te.pid.o’a.sis. L. masc. adj. *tepidus*, moderately warm; L. fem. n. *oasis*, oasis; N.L. gen. fem. n. *tepidoasis*, of a warm oasis, as the type strain was isolated from warm sites in a deep-sea hydrothermal vent field, often likened to an oasis in a deep-sea desert).

Gram-reaction-negative, motile, oval cells; sometimes appearing as cocci or plump rods. Cell size approximately 1.0–3.0 µm long and 0.8–1.5 µm wide. Possesses a single polar flagellum and a type I intracytoplasmic membrane system. Reproduces by normal cell division. Does not form cysts. Moderately thermophilic, growing at temperatures of 25–56 °C (optimum 45–50 °C) and at pH 5.2–6.9 (optimum pH 5.9–6.4). Requires 1.5–4 % (w/v) NaCl for growth (optimum 2–3 %). Grows aerobically on methane or methanol. Possesses particulate methane monooxygenase but no soluble methane monooxygenase. Assimilates C_1_ compounds via the RuMP pathway. Uses ammonium or nitrate as a nitrogen source. Does not fix atmospheric nitrogen for growth. Requires vitamins for growth. The major fatty acids are C_16 : 1_ω7*c*, C_16 : 0_ and C_18 : 1_ω7*c*. The major polar lipid is phosphatidylserine. The major isoprenoid quinone is Q-8.

The type strain is IN45^T^ (JCM 35101^T^ =DSM 113422^T^), which was isolated from *in situ* colonisation systems deployed at the Original site in the Iheya North deep-sea hydrothermal field in the mid-Okinawa Trough, Japan. The genome of the type strain consists of a 2.42-Mbp chromosome and a 20.5-kbp plasmid. The G+C content of the chromosomal DNA is 64.1 mol%.

The GenBank/EMBL/DDBJ accession numbers are LC770110 for the 16S rRNA gene sequence and AP024718 and AP024719 for the chromosome and plasmid sequence.

## supplementary material

10.1099/ijsem.0.006288 Uncited Supplementary Material 1.

## References

[1] Schubert CJ (2011). Methane, origin. Encycl Geobiol.

[2] Dubilier N, Bergin C, Lott C (2008). Symbiotic diversity in marine animals: the art of harnessing chemosynthesis. Nat Rev Microbiol.

[3] Dick GJ, Tebo BM (2010). Microbial diversity and biogeochemistry of the Guaymas basin deep-sea hydrothermal plume. Environ Microbiol.

[4] Distel DL, Lee HK, Cavanaugh CM (1995). Intracellular coexistence of methano- and thioautotrophic bacteria in a hydrothermal vent mussel. Proc Natl Acad Sci U S A.

[5] Hirayama H, Takaki Y, Abe M, Imachi H, Ikuta T (2022). Multispecies populations of methanotrophic *Methyloprofundus* and cultivation of a likely dominant species from the Iheya North deep-sea hydrothermal field. Appl Environ Microbiol.

[6] Nercessian O, Bienvenu N, Moreira D, Prieur D, Jeanthon C (2005). Diversity of functional genes of methanogens, methanotrophs and sulfate reducers in deep-sea hydrothermal environments. Environ Microbiol.

[7] Skennerton CT, Ward LM, Michel A, Metcalfe K, Valiente C (2015). Genomic reconstruction of an uncultured hydrothermal vent gammaproteobacterial methanotroph (family *Methylothermaceae*) indicates multiple adaptations to oxygen limitation. Front Microbiol.

[8] Hirayama H, Abe M, Miyazaki M, Nunoura T, Furushima Y (2014). *Methylomarinovum caldicuralii* gen. nov., sp. nov., a moderately thermophilic methanotroph isolated from a shallow submarine hydrothermal system, and proposal of the family *Methylothermaceae* fam. Int J Syst Evol Microbiol.

[9] Takeuchi M, Kamagata Y, Oshima K, Hanada S, Tamaki H (2014). *Methylocaldum marinum* sp. nov., a thermotolerant, methane-oxidizing bacterium isolated from marine sediments, and emended description of the genus *Methylocaldum*. Int J Syst Evol Microbiol.

[10] Hirayama H, Suzuki Y, Abe M, Miyazaki M, Makita H (2011). *Methylothermus subterraneus* sp. nov., a moderately thermophilic methanotroph isolated from a terrestrial subsurface hot aquifer. Int J Syst Evol Microbiol.

[11] Tsubota J, Eshinimaev BT, Khmelenina VN, Trotsenko YA (2005). *Methylothermus thermalis* gen. nov., sp. nov., a novel moderately thermophilic obligate methanotroph from a hot spring in Japan. Int J Syst Evol Microbiol.

[12] Heyer J, Berger U, Hardt M, Dunfield PF (2005). *Methylohalobius crimeensis* gen. nov., sp. nov., a moderately halophilic, methanotrophic bacterium isolated from hypersaline lakes of Crimea. Int J Syst Evol Microbiol.

[13] Rush D, Osborne KA, Birgel D, Kappler A, Hirayama H (2016). The bacteriohopanepolyol inventory of novel aerobic methane oxidising bacteria reveals new biomarker signatures of aerobic methanotrophy in marine systems. PLoS One.

[14] Lane DJ. (1991). 16S/23S Sequencing.

[15] Pruesse E, Peplies J, Glöckner FO (2012). SINA: accurate high-throughput multiple sequence alignment of ribosomal RNA genes. Bioinformatics.

[16] Trifinopoulos J, Nguyen L-T, von Haeseler A, Minh BQ (2016). W-IQ-TREE: a fast online phylogenetic tool for maximum likelihood analysis. Nucleic Acids Res.

[17] Kalyaanamoorthy S, Minh BQ, Wong TKF, von Haeseler A, Jermiin LS (2017). ModelFinder: fast model selection for accurate phylogenetic estimates. Nat Methods.

[18] Hoang DT, Chernomor O, von Haeseler A, Minh BQ, Vinh LS (2018). UFBoot2: improving the ultrafast bootstrap approximation. Mol Biol Evol.

[19] Chin C-S, Alexander DH, Marks P, Klammer AA, Drake J (2013). Nonhybrid, finished microbial genome assemblies from long-read SMRT sequencing data. Nat Methods.

[20] Ruby JG, Bellare P, Derisi JL (2013). PRICE: software for the targeted assembly of components of (meta) genomic sequence data. G3.

[21] Walker BJ, Abeel T, Shea T, Priest M, Abouelliel A (2014). Pilon: an integrated tool for comprehensive microbial variant detection and genome assembly improvement. PLoS One.

[22] Hyatt D, LoCascio PF, Hauser LJ, Uberbacher EC (2012). Gene and translation initiation site prediction in metagenomic sequences. Bioinformatics.

[23] Nawrocki EP, Eddy SR (2013). Infernal 1.1: 100-fold faster RNA homology searches. Bioinformatics.

[24] Lowe TM, Chan PP (2016). tRNAscan-SE On-line: integrating search and context for analysis of transfer RNA genes. Nucleic Acids Res.

[25] Meier-Kolthoff JP, Auch AF, Klenk H-P, Göker M (2013). Genome sequence-based species delimitation with confidence intervals and improved distance functions. BMC Bioinformatics.

[26] Lee I, Ouk Kim Y, Park S-C, Chun J (2016). OrthoANI: an improved algorithm and software for calculating average nucleotide identity. Int J Syst Evol Microbiol.

[27] Chaumeil P-A, Mussig AJ, Hugenholtz P, Parks DH (2022). GTDB-Tk v2: memory friendly classification with the genome taxonomy database. Bioinformatics.

[28] Stamatakis A (2014). RAxML version 8: a tool for phylogenetic analysis and post-analysis of large phylogenies. Bioinformatics.

[29] Edgar RC (2004). MUSCLE: multiple sequence alignment with high accuracy and high throughput. Nucleic Acids Res.

[30] Buck JD (1982). Nonstaining (KOH) method for determination of Gram reactions of marine bacteria. Appl Environ Microbiol.

[31] Steyer AM, Ruhwedel T, Möbius W (2019). Biological sample preparation by high-pressure freezing, microwave-assisted contrast enhancement, and minimal resin embedding for volume imaging. J Vis Exp.

[32] Deerinck TJ, Bushong EA, Thor A, Ellisman MH (2010). NCMIR Methods for 3D EM: A New Protocol for Preparation of Biological Specimens for Serial Block Face Scanning Electron Microscopy.

[33] Minnikin DE, O’Donnell AG, Goodfellow M, Alderson G, Athalye M (1984). An integrated procedure for the extraction of bacterial isoprenoid quinones and polar lipids. J Microbiol Met.

[34] Komagata K, Suzuki K-I, Colwell RR, Grigorova R (1988). Methods Microbiol.

[35] Sasser M (1990). MIDI Technical Note.

[36] Christie WW, Christie WW (1997). Advances in Lipid Methodology - Four.

[37] Kim M, Oh H-S, Park S-C, Chun J (2014). Towards a taxonomic coherence between average nucleotide identity and 16S rRNA gene sequence similarity for species demarcation of prokaryotes. Int J Syst Evol Microbiol.

[38] Goris J, Konstantinidis KT, Klappenbach JA, Coenye T, Vandamme P (2007). DNA–DNA hybridization values and their relationship to whole-genome sequence similarities. Int J Syst Evol Microbiol.

[39] Richter M, Rosselló-Móra R (2009). Shifting the genomic gold standard for the prokaryotic species definition. Proc Natl Acad Sci U S A.

[40] Konstantinidis KT, Rosselló-Móra R, Amann R (2017). Uncultivated microbes in need of their own taxonomy. ISME J.

[41] Chen KH-C, Wu H-H, Ke S-F, Rao Y-T, Tu C-M (2012). Bacteriohemerythrin bolsters the activity of the particulate methane monooxygenase (pMMO) in *Methylococcus capsulatus* (Bath). J Inorg Biochem.

[42] Nariya S, Kalyuzhnaya MG (2020). Hemerythrins enhance aerobic respiration in *Methylomicrobium alcaliphilum* 20ZR, a methane-consuming bacterium. FEMS Microbiol Lett.

[43] Koendjbiharie JG, Hon S, Pabst M, Hooftman R, Stevenson DM (2020). The pentose phosphate pathway of cellulolytic clostridia relies on 6-phosphofructokinase instead of transaldolase. J Biol Chem.

[44] Garschagen LS, Franke T, Deppenmeier U (2021). An alternative pentose phosphate pathway in human gut bacteria for the degradation of C5 sugars in dietary fibers. FEBS J.

[45] Nakahara K, Yamamoto H, Miyake C, Yokota A (2003). Purification and characterization of class-I and class-II fructose-1,6-bisphosphate aldolases from the cyanobacterium *Synechocystis* sp. PCC 6803. Plant Cell Physiol.

[46] Nakahigashi K, Toya Y, Ishii N, Soga T, Hasegawa M (2009). Systematic phenome analysis of *Escherichia coli* multiple-knockout mutants reveals hidden reactions in central carbon metabolism. Mol Syst Biol.

[47] Reshetnikov AS, Rozova ON, Khmelenina VN, Mustakhimov II, Beschastny AP (2008). Characterization of the pyrophosphate-dependent 6-phosphofructokinase from *Methylococcus capsulatus* Bath. FEMS Microbiol Lett.

[48] Rozova ON, Khmelenina VN, Mustakhimov II, Reshetnikov AS, Trotsenko YA (2010). Characterization of recombinant fructose-1,6-bisphosphate aldolase from *Methylococcus capsulatus* bath. Biochemistry.

[49] Rozova ON, Khmelenina VN, Trotsenko YA (2012). Characterization of recombinant PPi-dependent 6-phosphofructokinases from *Methylosinus trichosporium* OB3b and *Methylobacterium nodulans* ORS 2060. Biochemistry.

[50] Stolzenberger J, Lindner SN, Persicke M, Brautaset T, Wendisch VF (2013). Characterization of fructose 1,6-bisphosphatase and sedoheptulose 1,7-bisphosphatase from the facultative ribulose monophosphate cycle methylotroph *Bacillus methanolicus*. J Bacteriol.

[51] Fan L, Wang Y, Qian J, Gao N, Zhang Z (2021). Transcriptome analysis reveals the roles of nitrogen metabolism and sedoheptulose bisphosphatase pathway in methanol-dependent growth of *Corynebacterium glutamicum*. Microb Biotechnol.

[52] Woolston BM, King JR, Reiter M, Van Hove B, Stephanopoulos G (2018). Improving formaldehyde consumption drives methanol assimilation in engineered *E. coli*. Nat Commun.

[53] Augimeri RV, Varley AJ, Strap JL (2015). Establishing a role for bacterial cellulose in environmental interactions: lessons learned from diverse biofilm-producing proteobacteria. Front Microbiol.

[54] Römling U, Galperin MY (2015). Bacterial cellulose biosynthesis: diversity of operons, subunits, products, and functions. Trends Microbiol.

[55] Kawagucci S, Miyazaki J, Nakajima R, Nozaki T, Takaya Y (2013). Post‐drilling changes in fluid discharge pattern, mineral deposition, and fluid chemistry in the Iheya North hydrothermal field, Okinawa Trough. Geochem Geophys Geosyst.

[56] Balch WE, Fox GE, Magrum LJ, Woese CR, Wolfe RS (1979). Methanogens: reevaluation of a unique biological group. Microbiol Rev.

[57] Kuwamoto K (2019). Simple and quick quality confirmation method of aqueous urea solution for marine SCR system: evaluation of alkalinity of aqueous urea solution with Laquatwin pH meter. Readout.

[58] Skiba U, Jørgensen SE, Fath BD (2008). Encyclopedia of Ecology.

[59] Hoefman S, van der Ha D, Boon N, Vandamme P, De Vos P (2014). Niche differentiation in nitrogen metabolism among methanotrophs within an operational taxonomic unit. BMC Microbiol.

[60] Kits KD, Klotz MG, Stein LY (2015). Methane oxidation coupled to nitrate reduction under hypoxia by the Gammaproteobacterium *Methylomonas denitrificans*, sp. nov. type strain FJG1. Environ Microbiol.

