# Supplementary material for: Methylomarinovum tepidoasis sp. nov., a moderately thermophilic methanotroph of the family Methylothermaceae isolated from a deep-sea hydrothermal field
Source: Int J Syst Evol Microbiol. 2024 Mar 13;74(3):006288. doi: 10.1099/ijsem.0.006288 (PMC10950024; doi:10.1099/ijsem.0.006288)
Supplement: Uncited Supplementary Material 1. [file ijsem-74-06288-s001.pdf]

Supplementary Materials

Table S1 List of selected genes for central carbon and nitrogen metabolism

Description	EC Number	Gene	Strain IN45^T Locus Tag	*Methylomarinovum caldicuralii* IT-9^T Locus Tag
**Methane oxidation**				
methane monooxygenase subunit C	1.14.18.3	pmoC	MIN45_P0549	MIT9_P0453
methane monooxygenase subunit A	1.14.18.3	pmoA	MIN45_P0548	MIT9_P0452
methane monooxygenase subunit B	1.14.18.3	pmoB	MIN45_P0547	MIT9_P0451
methane monooxygenase subunit C	1.14.18.3	pmoC	MIN45_P2285	MIT9_P0922
methane monooxygenase subunit A	1.14.18.3	pmoA	MIN45_P2284	MIT9_P0923
methane monooxygenase subunit B	1.14.18.3	pmoB	MIN45_P2283	MIT9_P0924
methane monooxygenase subunit C (orphan)	1.14.18.3	pmoC	MIN45_P0884	MIT9_P0642
			MIN45_P1705	MIT9_P1976
**Methanol oxidation**				
methanol dehydrogenase subunit 1	1.1.2.7	mxaF	MIN45_P1622	MIT9_P2082
methanol dehydrogenase subunit 2	1.1.2.7	mxaI	MIN45_P1625	MIT9_P2079
lanthanide-dependent methanol dehydrogenase	1.1.2.10	xoxF	MIN45_P1657	MIT9_P2026
**Formaldehyde oxidation (tetrahydromethanopterin-mediated C1 transfer) to CO_2**				
5,6,7,8-tetrahydromethanopterin hydro-lyase	4.2.1.147	fae	MIN45_P1340	MIT9_P1293
			MIN45_P1844	MIT9_P1444
			MIN45_P2222	MIT9_P1772
methylene-tetrahydromethanopterin dehydrogenase	1.5.1.-	mtdB	MIN45_P0900	MIT9_P0625
			MIN45_P0901	MIT9_P0624
methenyltetrahydromethanopterin cyclohydrolase	3.5.4.27	mch	MIN45_P1841	MIT9_P1441
formylmethanofuran--tetrahydromethanopterin N-formyltransferase	2.3.1.101	ffsA	MIN45_P1471	MIT9_P1920
formylmethanofuran dehydrogenase subunit B	1.2.7.12	fhcB, fwdB	MIN45_P1468	MIT9_P1923
formylmethanofuran dehydrogenase subunit A	1.2.7.12	fhcA, fwdA	MIN45_P1470	MIT9_P1921
				MIT9_P1576
formylmethanofuran dehydrogenase subunit C	1.2.7.12	fhcC, fwdC	MIN45_P1472	MIT9_P1919
formate dehydrogenase major subunit	1.17.1.9	fdoG, fdhF, fdwA	MIN45_P0076	MIT9_P0955
formate dehydrogenase iron-sulfur subunit		fdoH, fdsB	MIN45_P0075	MIT9_P0956
formate dehydrogenase subunit gamma		fdoI, fdsG	MIN45_P0074	MIT9_P0957

formate dehydrogenase major subunit	1.17.1.9	fdoG, fdhF, fdwA	MIN45_P1656	MIT9_P2027
formate dehydrogenase beta subunit	1.17.1.9	fdwB	MIN45_P1655	MIT9_P2028
**Tetrahydrofolate-mediated C1 transfer pathway**				
formate--tetrahydrofolate ligase	6.3.4.3	fhs	MIN45_P0550	MIT9_P0454
methenyltetrahydrofolate cyclohydrolase	3.5.4.9	fchA	MIN45_P1255	MIT9_P1268
methylenetetrahydrofolate/methylenetetrahydromethanopterin dehydrogenase (NADP+)	1.5.1.5	mtdA	MIN45_P1256	MIT9_P1269
glycine hydroxymethyltransferase	2.1.2.1	glyA	MIN45_P1981	MIT9_P1388
**Ribulose monophosphate (RuMP) pathway**				
3-hexulose-6-phosphate synthase	4.1.2.43	hxlA, hps	MIN45_P0982	MIT9_P2554
6-phospho-3-hexuloisomerase	5.3.1.27	hxlB, phi	MIN45_P0983	MIT9_P2553
ATP-dependent phosphofructokinase/ diphosphate-dependent phosphofructokinase	2.7.1.11, 2.7.1.90	pfk, pfp	MIN45_P1835	MIT9_P1487
6-phosphofructokinase 2	2.7.1.11	pfkB	MIN45_P2011	MIT9_P1357
fructose-1,6-bisphosphatase I	3.1.3.11	fbp	MIN45_P1403	MIT9_P2137
fructose-bisphosphate aldolase, class II	4.1.2.13	fba, fbaA	MIN45_P1405<br>MIN45_P1406	MIT9_P2134<br>MIT9_P2135
transketolase	2.2.1.1	tkt	MIN45_P1404	MIT9_P2136
ribose 5-phosphate isomerase A	5.3.1.6	rpiA	MIN45_P0705	MIT9_P0662<br>MIT9_P1520
ribulose-phosphate 3-epimerase	5.1.3.1	rpe	MIN45_P0817<br>MIN45_P1814	MIT9_P0835<br>MIT9_P1259
glucose-6-phosphate isomerase	5.3.1.9	pgi	MIN45_P1440	MIT9_P0005
glucose-6-phosphate 1-dehydrogenase	1.1.1.49, 1.1.1.363	zwf	MIN45_P0734	MIT9_P0692
6-phosphogluconolactonase	3.1.1.31	pgl	MIN45_P1441	MIT9_P0004
6-phosphogluconate dehydrogenase	1.1.1.44, 1.1.1.343	gnd	MIN45_P1895	MIT9_P1472
**Embden–Meyerhof–Parnas (EMP) pathway**				
triosephosphate isomerase	5.3.1.1	tpiA	MIN45_P2281	MIT9_P0448
glyceraldehyde 3-phosphate dehydrogenase	1.2.1.12	gapA	MIN45_P1401	MIT9_P2139
phosphoglycerate kinase	2.7.2.3	pgk	MIN45_P1681	MIT9_P2002
2,3-bisphosphoglycerate-dependent phosphoglycerate mutase	5.4.2.11	PGAM, gpmA	MIN45_P0103	MIT9_P1789
enolase	4.2.1.11	eno	MIN45_P0168	MIT9_P1876
pyruvate kinase	2.7.1.40	pyk	MIN45_P1402<br>MIN45_P2052	MIT9_P1334<br>MIT9_P2138

pyruvate, water dikinase	2.7.9.2	pps	MIN45_P0034<br>MIN45_P1485	MIT9_P1070<br>MIT9_P2189
**TCA cycle**				
pyruvate dehydrogenase E1 component	1.2.4.1	aceE	MIN45_P1452	MIT9_P0992
pyruvate dehydrogenase E2 component (dihydrolipoamide acetyltransferase)	2.3.1.12	aceF	MIN45_P1453	MIT9_P0993
dihydrolipoamide dehydrogenase	1.8.1.4	lpd	MIN45_P1454	MIT9_P0994
pyruvate dehydrogenase E1 component alpha subunit	1.2.4.1	pdhA	MIN45_P2060	MIT9_P1327
pyruvate dehydrogenase E1 component beta subunit	1.2.4.1	pdhB	MIN45_P2061	MIT9_P1326
pyruvate dehydrogenase E2 component (dihydrolipoamide acetyltransferase)	2.3.1.12	pdhC, aceF	MIN45_P2062	MIT9_P1325
pyruvate carboxylase subunit A	6.4.1.1	pycA	MIN45_P1107	MIT9_P2390
pyruvate carboxylase subunit B	6.4.1.1	pycB	MIN45_P1106	MIT9_P2391
carboxybiotin decarboxylase	7.2.4.1	oadB	MIN45_P1779	MIT9_P1607
oxaloacetate decarboxylase (Na+ extruding) subunit alpha	7.2.4.2	oadA	MIN45_P1780	MIT9_P1606
oxaloacetate decarboxylase (Na+ extruding) subunit gamma		oadG	MIN45_P1781	MIT9_P1605
citrate synthase	2.3.3.1	gltA	MIN45_P1390<br>MIN45_P2147	MIT9_P2104<br>MIT9_P2166
aconitate hydratase	4.2.1.3	acnA	MIN45_P1752	MIT9_P1633
isocitrate dehydrogenase (NAD+)	1.1.1.41	IDH3	MIN45_P1339	MIT9_P1292
2-oxoglutarate dehydrogenase E1 component	1.2.4.2	sucA	MIN45_P1296	MIT9_P1277
2-oxoglutarate dehydrogenase E2 component	2.3.1.61	sucB	MIN45_P1295	MIT9_P1276
succinyl-CoA synthetase alpha subunit	6.2.1.5	sucD	MIN45_P0273<br>MIN45_P1850	MIT9_P1451<br>MIT9_P2277
succinyl-CoA synthetase beta subunit	6.2.1.5	sucC	MIN45_P0274<br>MIN45_P1851	MIT9_P1452<br>MIT9_P2278
succinate dehydrogenase, iron-sulfur subunit	1.3.5.1	sdhB	MIN45_P2312	MIT9_P0895
succinate dehydrogenase, flavoprotein subunit	1.3.5.1	sdhA	MIN45_P2313	MIT9_P0894
succinate dehydrogenase, membrane anchor subunit		sdhD	MIN45_P2314	MIT9_P0893
succinate dehydrogenase, cytochrome b subunit		sdhC	MIN45_P2315	MIT9_P0892
fumarate hydratase, class II	4.2.1.2	fumC	MIN45_P1007	MIT9_P2523
malate dehydrogenase	1.1.1.37	mdh	MIN45_P0471	MIT9_P0372
**Denitrification**				
MFS transporter, NNP family, nitrate/nitrite transporter		NRT, narK	MIN45_P0036	–
nitrate reductase/nitrite oxidoreductase, alpha subunit	1.7.5.1	narG	MIN45_P0037	–

nitrate reductase/nitrite oxidoreductase, beta subunit	1.7.5.1	narH	MIN45_P0038	–
nitrate reductase molybdenum cofactor assembly chaperone		narJ	MIN45_P0039	–
nitrate reductase gamma subunit	1.7.5.1	narI	MIN45_P0040	–
nitrite reductase (NO-forming)	1.7.2.1	nirK	MIN45_P0203	–
nitric oxide reductase subunit B	1.7.2.5	norB	MIN45_P1955	–
nitric oxide reductase subunit C		norC	MIN45_P1956	–
**Other nitrogen metabolism**				
assimilatory nitrate reductase catalytic subunit		nasA	MIN45_P0415	–
MFS transporter, NNP family, nitrate/nitrite transporter		NRT, narK	MIN45_P0416	–
nitrite reductase (NADH) large subunit	1.7.1.15	nirB	MIN45_P0412	–
nitrite reductase (NADH) small subunit	1.7.1.15	nirD	MIN45_P0413	–
ammonium transporter, Amt family		amt	MIN45_P0081<br>MIN45_P0408	MIT9_P0315<br>MIT9_P0891
glutamine synthetase	6.3.1.2	glnA	MIN45_P0403	MIT9_P0310
glutamate dehydrogenase	1.4.1.2	GDH2	MIN45_P2038	–
hydroxylamine dehydrogenase	1.7.2.6	hao	MIN45_P1027	MIT9_P2476
**Glycogen synthesis**				
starch synthase	2.4.1.21	glgA	MIN45_P1344	MIT9_P1297
1,4-alpha-glucan branching enzyme	2.4.1.18	glgB	MIN45_P1345	MIT9_P1298
glucose-1-phosphate adenylyltransferase	2.7.7.27	glgC	MIN45_P1346	MIT9_P1299
**Celluose synthesis**				
cellulose synthase (UDP-forming)	2.4.1.12	bcsA	MIN45_P1066	MIT9_P2436 (disrupted)
cellulose synthase operon protein B		bcsB	MIN45_P1065	MIT9_P2438
endoglucanase	3.2.1.4	bcsZ	MIN45_P1064	MIT9_P2439
cellulose synthase operon protein C		bcsC	MIN45_P1063	MIT9_P2440
**Others**				
flagellin		fliC	MIN45_P1444<br>MIN45_P1445	MIT9_P0983
hemerythrin			MIN45_P1762	–

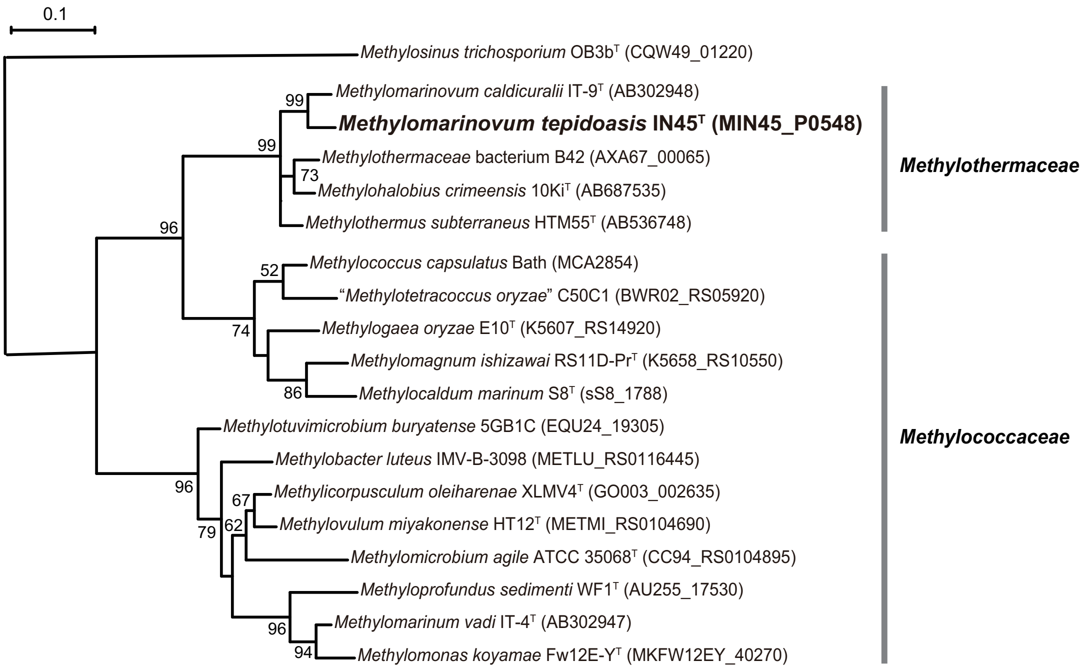

Supplementary Fig. S1

Maximum likelihood phylogenetic tree based on deduced full-length PmoA sequences (254 amino acid positions). Bootstrap analysis (1000 replicates) was performed and only bootstrap values >50% are shown at nodes. Accession numbers or gene locus tags are indicated in parentheses. *Methylosinus trichosporium* OB3b^{T} was used as an outgroup species.

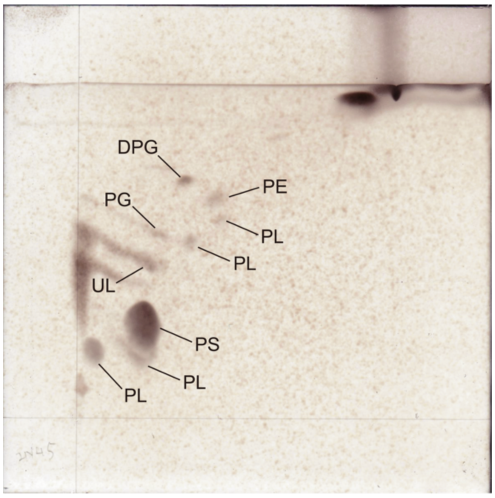

Supplementary Fig. S2

Polar lipid profile of strain IN45^{T} by two-dimensional thin-layer chromatography. DPG, diphosphatidylglycerol; PE, phosphatidylethanolamine; PG, phosphatidylglycerol; PL, unknown phospholipids; PS, phosphatidylserine; UL, unknown lipids

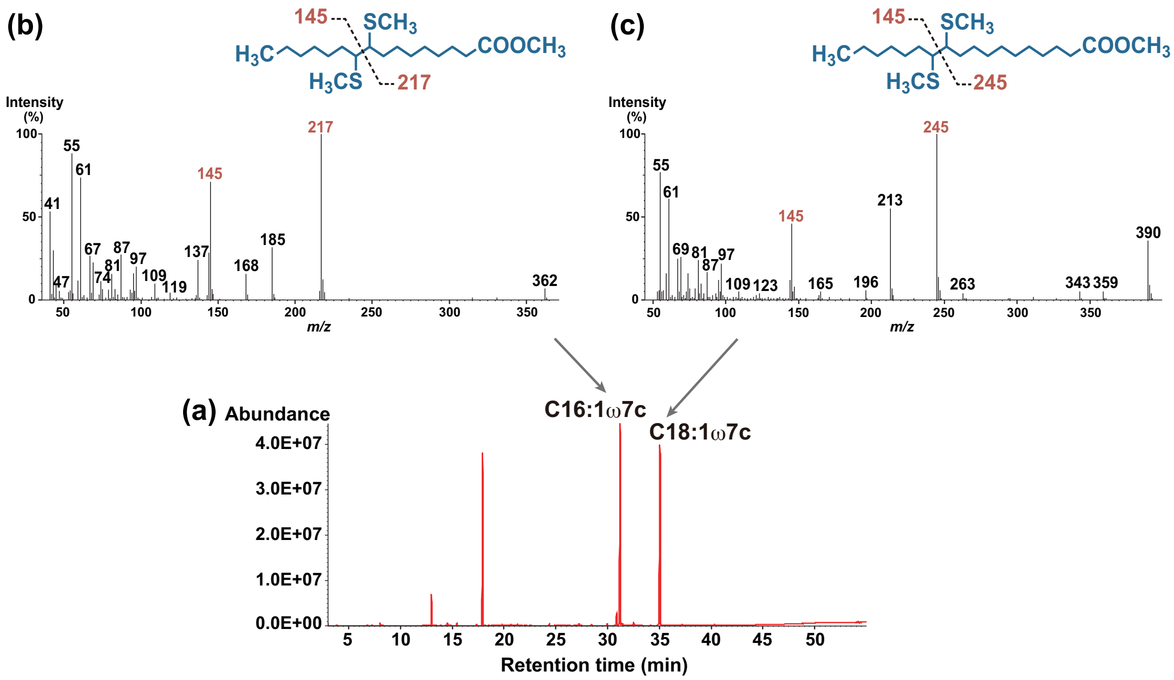

Supplementary Fig. S3

Determination of double bond positions in unsaturated fatty acids from strain IN45[T]. (a) Gas chromatogram of dimethyl disulfide derivatives of fatty acid methyl esters. (b, c) Mass spectra of $C_{16:1}$ ω7c (b) and $C_{18:1}$ ω7c (c).
